# Supplementary material for: Feature engineering and machine learning for computer-assisted screening of children with speech disorders
Source: PLOS Digit Health. 2022 May 26;1(5):e0000041. doi: 10.1371/journal.pdig.0000041 (PMC9931328; doi:10.1371/journal.pdig.0000041)
Supplement: Attachment — Submitted filename: Response to Reviewers.docx [file pdig.0000041.s001.docx]

**Reviewer #1: The authors proposed a computer-assisted approach for children speech disorders screening. They used the SpeechMark software to extract speech landmarks as features, based on which some standard feature engineering strategies (e.g., n-gram) and feature selection methods were applied. They also employed SMOTE method to handle the class imbalance issue. Standard machine learning algorithms (e.g., LDA, SVM, XGBoost, RF) were then trained based on those engineered features. The methods in this work are generally sound and they showed significant improvement in model performance with their feature engineering and selection strategies. However, there are a few concerns as listed below.**

**1. It is not clear if the authors had a separate testing set. It seemed that they used cross-validation or leave-one-out methods for feature selection, handled sample imbalance, and tuned hyper-parameters, all on the whole dataset. If they did not have a testing set separated before employing all the engineering and tuning, the models would likely be overfitted. This does not show in current results but will show when applied to new data that the models have not seen. The authors should at least try to separate a testing set of about 20% of the data, do all the feature engineering and training on the rest, and test the models on the testing set.**

**Authors’ response:** First of all, we want to thank the reviewer for the insightful and constructive comments. We have carefully addressed each of them below and incorporated them into the revision to help improve the clarity of the paper.

1. All the results presented in the paper are based on the unseen test data (*i.e.*, they’re not involved in any training steps such as feature selection or hyper-parameter tuning).

Due to the small number of samples, we would not have enough data for model training if we had taken 20% of the dataset for the test set. As a result, we decided to take 1 sample from each class for the test set (i.e., 2 samples in the testing set). To avoid bias or cherry-picking due to the small number of the test samples, we performed 50 Monte Carlo cross-validation and testing (MCVT). In each MCVT, we randomly select two samples for the test set and set them aside; then, we perform the SMOTE on the training set. For both feature selection and hyperparameter tuning, only samples from the training set were used. For feature selection, 5-fold recursive feature elimination with cross-validation (RFECV) was used. For hyperparameter tuning, we used 10-fold cross-validation, again with only samples from the training set. This complete separation of training from testing is also shown in Figure 3. For evaluating the performance of our classification models, we take the mean of 50 MCVTs and report them, again, to provide a fair evaluation instead of cherry picking. More specifics are provided below:

For handling class imbalance, we have used SMOTE. But it was performed only on the training set. The code’s name for this part is: “Data_generation_50SMOTE”. The part where we separate the train and test is shown below:

After splitting the training and test sets, we performed SMOTE only on the training set, and this part is also shown here:

The hyperparameter tuning is done on the training set using 10-fold cross-validation for every classification technique we use. An example of this is brought below, which is a part of this code: “LDA_SVD_10_selected_features”.

It is worth noting that the process of selecting the 10 features was also done only on the training set. The code for this part is in file: “LDA_RFECV_189features”.

To improve the clarity on this point, we have incorporated the above discussions in the revision, especially in the Results and discussion, and the Conclusion sections.
**2. Another shortcoming of this work is the limited data size – only from 51 children, which makes it hard to draw any significant conclusions.**

**Authors’ response:** Small sample size has always been a limitation in biomedical studies due to labor, time and other constraints. The drawback of having limited data for studies related to children’s speech has been reported before in other studies [1]. However, we respectfully disagree with the reviewer that this study (based on 51 samples) cannot draw any significant conclusions. First, Monte Carlo validation and testing (MCVT), which is based on Monte Carlo cross-validation (MCCV), is used to overcome this limitation. The training and testing sets are randomly selected in each outer MC loop based on the proportions specified (see Fig. 3). Then, the training set splits into training and validation sets in each inner cross-validation MC run. Finally, this method gets the average of performance metrics from different runs [2]. We repeat this loop 50 times. This method provides fair evaluations even with small number of samples. We also used feature selection (using training samples only) to avoid overfitting and reduce prediction variance. Finally, we utilized SMOTE to generate synthetic samples (again, in training set only) to help alleviate the problem as well. Nevertheless, the reviewer had a valid point, and we didn’t make our procedure clear enough in our original submission. We’ve now incorporated the above discussions into the revision (in both Results and discussion section and Conclusion section).

**3. A related work using SpeechMark for landmarks extraction and machine learning algorithms for depression detection should be cited and compared.** [**https://ieeexplore-ieee-org.ezproxy.lib.nctu.edu.tw/abstract/document/8682916**](https://ieeexplore-ieee-org.ezproxy.lib.nctu.edu.tw/abstract/document/8682916)

**Authors response:** Thank you for the comment. We have included this work in the introduction section. With novel features proposed, our approach achieves better results than this work.

**A few minor points:

1. There are duplicated references: 18 and 21, 19 and 22.**

**Authors response:** Thank you for pointing it out. We corrected the references.

**2. Figure 8 is not a standard ROC curve. Please refer to Figure 4 and link the left-most point to the origin and the right-most point to the top-right corner. Do not plot with circles. May consider labeling data values so the readers can see clearly the key points.**

**Authors response:** This figure has been updated. Thank you for pointing it out.

References:

[1] Leitao, S. and Fletcher, J., 2004. Literacy outcomes for students with speech impairment: long‐term follow‐up. International Journal of Language & Communication Disorders, 39(2), pp.245-256.

[2] Shah, D., Wang, J. and He, Q.P., 2019. A feature-based soft sensor for spectroscopic data analysis. Journal of Process Control, 78, pp.98-107.
